# Osteopontin and oral carcinogenesis

**DOI:** 10.1111/j.1582-4934.2008.00348.x

**Published:** 2008-04-15

**Authors:** Shailendra Kapoor

**Affiliations:** University of Illinois at ChicagoChicago, IL, USA

Dear Editor:

The article by Chakraborty *et al*. about the relationship between down-regulation of osteopontin and breast tumour progression *in vivo*is highly interesting [1]. Recent studies indicate a close association between not only breast cancer and osteopontin but other systemic malignancies as well. Studies over the past few years have especially shown a strong relationship between osteopontin expression and oral carcinogenesis.

For instance, Devoll *et al*. in a recent study have reported that nearly 67% of oral squamous cell carcinomas and nearly 54% of all oral carcinomas *in situ* lesions are immunoreactive for osteopontin [2]. Similarly, a recent study by Matsuzaki *et al*. suggests that osteo-pontin may be an important marker of early invasion in lingular squamous cell carcinomas [3]. In fact, tumours of the tongue that overexpress osteopontin are usually more aggressive and carry a worse prognosis [4]. It has also been shown that osteopontin expression is increased in salivary gland tumours especially in polymorphous low-grade adenocarcinomas and pleomorphic adenomas [5]. More recently, antisense oligonucleotides have been used to inhibit tumerogenesis in oral cancer cell lines further confirming the pathological role of osteopontin in oral carcinogenesis [6].

Besides oral malignancies, osteopontin has also been implicated in the etio-pathogenesis of numerous other systemic malignancies such as non-small cell lung cancers and breast cancers [7]. Clearly, osteopontin has a major role to play in the development and growth of oral malignancies. Research studies should focus on osteopontin as a possible target molecule for the development of strategies and therapies to control oral cancers.
